# Low-Level Information and High-Level Perception: The Case of Speech in Noise 

**DOI:** 10.1371/journal.pbio.0060126

**Published:** 2008-05-20

**Authors:** Mor Nahum, Israel Nelken, Merav Ahissar

**Affiliations:** 1 Interdisciplinary Center for Neural Computation (ICNC), Hebrew University, Jerusalem, Israel; 2 Department of Neurobiology, Hebrew University, Jerusalem, Israel; 3 Department of Psychology, Hebrew University, Jerusalem, Israel; University of Maryland College Park, United States of America

## Abstract

Auditory information is processed in a fine-to-crude hierarchical scheme, from low-level acoustic information to high-level abstract representations, such as phonological labels. We now ask whether fine acoustic information, which is not retained at high levels, can still be used to extract speech from noise. Previous theories suggested either full availability of low-level information or availability that is limited by task difficulty. We propose a third alternative, based on the Reverse Hierarchy Theory (RHT), originally derived to describe the relations between the processing hierarchy and visual perception. RHT asserts that only the higher levels of the hierarchy are immediately available for perception. Direct access to low-level information requires specific conditions, and can be achieved only at the cost of concurrent comprehension. We tested the predictions of these three views in a series of experiments in which we measured the benefits from utilizing low-level binaural information for speech perception, and compared it to that predicted from a model of the early auditory system. Only auditory RHT could account for the full pattern of the results, suggesting that similar defaults and tradeoffs underlie the relations between hierarchical processing and perception in the visual and auditory modalities.

## Author Summary

One of the central questions in sensory neuroscience is the determination of the maximal amount of task-relevant information that is encoded in our brain. It is often assumed that all of this information is available for making perceptual decisions. We now show that this assumption does not hold generally. We find that when discriminating or understanding speech masked by noise, only the information that is represented at higher cortical areas is generally accessible for perception. Thus, when we need to decide whether the speaker said “day” or “night,” we are likely to succeed in this discrimination. However, when fine discriminations are required (e.g., “day” vs. “bay”), the information regarding the fine spectral and temporal details, which are necessary to discriminate these two words, can be fully utilized only under special conditions. These conditions include, for example, systematic repetitions of the stimuli, as often done in psychoacoustic experiments, or when one eliminates the need for comprehension and focuses on mere identification. These conditions are nonecological, and are not afforded in most daily situations.

## Abstract 3

The ability to both perceive and understand speech accurately depends on the loss of information along the acoustic-to-phonological hierarchy of auditory processing. A general theory of hierarchical processing in the brain predicts the conditions under which we can or cannot do both.

## Introduction

It is commonly accepted that auditory information is processed along the auditory pathways in a hierarchical manner [1–8], as in other sensory systems [9,10]. Although the functions of the various stages of this hierarchy, particularly at its cortical levels, are not well understood, the auditory hierarchy can be crudely divided into lower and higher representation levels [1,4,8]. Lower level representations reliably and selectively encode fine spectrotemporal acoustic features. Thus, at the brainstem level of the superior olivary complex (SOC), inputs from the two ears are compared within narrow frequency bands and with microsecond resolution [11–16]. In contrast, cortical levels integrate across time and frequency, and form more abstract, spectrotemporally broader, categories [5–8,17–22]. One of these higher representation levels is believed to be the phonological representation that underlies human speech perception [23–31]. A crucial property of these higher levels is the fact that acoustically different stimuli may belong to the same category (e.g., different instances of /ba/), whereas acoustically more similar stimuli may belong to different categories (e.g., similar instances of /ba/ and /da/) [20].

The fact that fine acoustic differences may be encoded at low levels of the auditory hierarchy, but not at its high levels, raises the question of whether such differences can be utilized for perceptual discriminations even when they are lost at the high representation levels. Although this question addresses the basic relations between the information available to the auditory system and our ability to use it for conscious perception, it is still unresolved. At least two different answers have been previously suggested.

A vast body of psychoacoustic studies proposes that all the information represented in the low levels of the auditory system is available for perception (termed here the *unlimited* view). Thus, perception fully utilizes low-level information, which is limited only by the variability of the neuronal responses at lower representation levels [32–37]. This claim is based on the ability of “ideal listener” models to account for human performance in a broad range of psychoacoustical tasks (e.g., [16,38–51]). These models usually assume two basic processing stages: a low-level neuronal representation of the input, which encapsulates all the information believed to be available at the level of the brainstem (e.g., [52]), and a subsequent decision-making stage [37], which performs statistically optimal decisions based on the full array of low-level activity [37].

On the other hand, a separate body of literature proposes that the answer depends on the behavioral context. Attention studies demonstrate that under demanding behavioral conditions, performance is lower than expected based on the information available at the low representation levels (e.g., [53–57]), i.e., poorer than the ideal listener prediction. Though most of these studies were conducted in the visual modality, there are also some compelling examples in the auditory domain. Particularly strong illustrations are provided by masking studies in which the stimuli are designed so that the low-level representations of the target and the masker do not overlap. Still, performance of many listeners is substantially degraded by the masking stimulus, indicating poor use of low-level information (“informational masking” [58–63]). Recent conceptualization of attention studies (e.g., [57,64–68]) defines demanding behavioral conditions in terms of the “load” they pose on the limited attentional and perceptual resources. These “limited-capacity” models, therefore, predict that under low attentional load, low-level information can be fully utilized, whereas under high load, the perceptual system can only process a portion of the relevant low-level information. The term *load* is not accurately defined, but is intuitively associated with task difficulty. Thus, as long as task difficulty remains the same, the ability to utilize low-level information should not change.

We now propose that the ability to use all the available low-level information depends on the stimulation protocol, rather than on the behavioral difficulty per se. This proposal is derived from the Reverse Hierarchy Theory (RHT), originally developed to address the relations between hierarchical processing and perception in the visual modality [69,70]. RHT had been successful in accounting for the discrepancy between the accurate spatial information available at lower levels of the visual hierarchy and its limited use in fast perceptual discriminations. We now apply its concepts to the auditory domain. According to RHT, high-level representations (such as phonological representations in the auditory domain) are immediately accessible to perception and therefore underlie our initial perceptual experience. Low-level representations (such as high-resolution interaural time differences) are accessible only under specific, privileged conditions. Hence, in general, low-level information would be available for perceptual discriminations only when high-level representations are essentially equivalent to the low-level representations. When this equivalence fails, perceptual discriminations can fully benefit from low-level resolution only under special behavioral conditions, which allow a search backwards along the “reverse hierarchy” for tracking the most informative low-level population.

In order to critically test the predictions of these three views, we measured the utilization of low-level information when extracting speech from noise, in a variety of behavioral conditions, which were administered in two studies. We calculated the expected ideal listener performance in each of these conditions. According to the “unlimited” view, performance should match ideal listener thresholds in all conditions. According to the “limited capacity” view, utilization of low-level information should depend on task difficulty (Study 2; Table 2) and would not change when task difficulty remains the same (Study 1; Table 1).

**Table 1:**
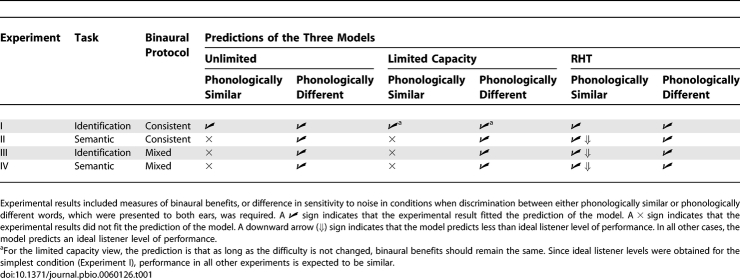
The Success of the Predictions of the Three Models (Unlimited, Limited Capacity, and Reverse Hierarchy Theory) for Experiments I–IV of Study 1

**Table 2:**
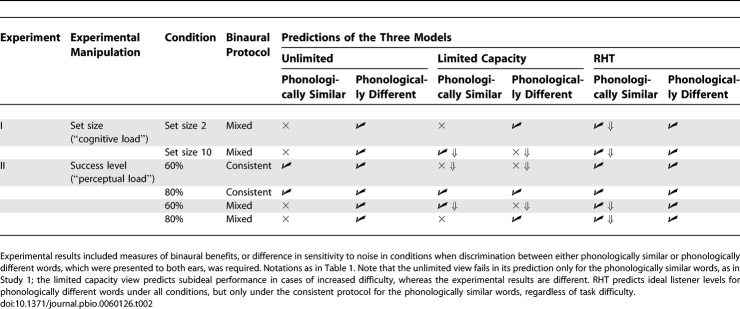
The Success of the Predictions of the Three Models (Unlimited, Limited Capacity, and Reverse Hierarchy Theory) for Experiments I and II of Study 2

In order to assess RHT predictions, we used two types of word sets, composed of phonologically different and phonologically similar words, respectively. This distinction is irrelevant for the ability of listeners to use low-level cues according to either the unlimited or the limited capacity views. However, RHT makes specific predictions for these two cases. Phonologically different words have distinctive low-level representations (since they are acoustically different) and distinctive high-level representations (since they are phonologically different). Thus, phonologically different words have the property that low-level and high-level representations are equivalent, and therefore, RHT predicts full use of low-level information, regardless of task difficulty (right-most column in Tables 1 and 2). In contrast, phonologically similar words have distinctive low-level representations (as will be demonstrated below for the word pairs used in this study), but at the phonological level, their representations will have a high degree of overlap (since they are phonologically similar). In this case, extracting the more abstract phonological categories causes partial loss of low-level information at the higher representation levels. Therefore, RHT predicts that the benefit from low-level information should match the performance predicted by ideal listener models only in specific protocols that allow backward search to find the informative low-level populations.

In the two studies we conducted, our measure for utilization of low-level information was the ability to use fine temporal cues between the inputs reaching the two ears in order to extract speech from noise (e.g., [13,44]). In ecological conditions, such time differences may arise when the source of the noise has a different azimuth than the source of the speech. Such time differences, which in humans are less than 1 ms, are usually expressed as phase differences since they are calculated within narrow frequency bands at the SOC [13,16,44]. We thus measured performance under two configurations of interaural phase differences, diotic and dichotic. The diotic configuration contains no phase information, since identical input (signal + noise) is presented to the two ears. The dichotic configuration maximizes phase information for separation between signal and noise [45,71]: the noise is identical in the two ears, while the signal is added with opposite phase to the two ears. The ability of listeners to use the low-level phase information was measured by the difference between dichotic and diotic thresholds (termed *binaural benefit*, typically in the range of 3–7 dB, e.g., [38,45,51,71–74]). Task difficulty was measured by diotic thresholds.

We found that human performance does not consistently match that of the ideal listener, in contrast to the unlimited view. However, task difficulty per se does not affect the ability to use low-level information, in contrast to the limited capacity view. Low-level information is always fully utilized when phonologically different words are used, but only under one specific protocol when phonologically similar words are used. This pattern matches the predictions of RHT, suggesting that its concept, of reversed relations between the hierarchy of processing and perceptual accessibility, is also applicable to the auditory modality.

## Results

The auditory stimuli we used were disyllabic Hebrew words and non-words embedded in speech noise [75], presented under both diotic and dichotic configurations (see Materials and Methods). All experiments were administered with both phonologically different word pairs (e.g., /tamid/ and /chalom/), and with phonologically similar word pairs, which differed in only one phoneme (e.g., /tamid/ and /amid/).

### Study 1—Manipulating Task Requirements while Retaining Its Difficulty

In this series of experiments, we asked whether binaural benefits can be modified without changing task difficulty, namely, without changing diotic thresholds. We used an ideal listener model (see Materials and Methods and Text S1) to calculate the expected performance. The two free parameters of the model (noise levels in the energy and correlation channels, respectively) were calculated from performance with a single, different set of words (used in Experiment II of Study 2). Thus, in all the calculations for this study, the model had no free parameters.

### Experiment I—Word Identification with No Binaural Uncertainty

The first experiment was designed to replicate studies that found binaural benefits to match those calculated by ideal listener models. The behavioral task was to identify which of the two words comprising the stimulus set (either phonologically different or phonologically similar) was presented in a given trial. Diotic and dichotic configurations were administered in separate blocks, so that the same binaural configuration was repeated across trials for each threshold measurement (“consistent”), eliminating binaural uncertainty within a block.

**Figure 1:**
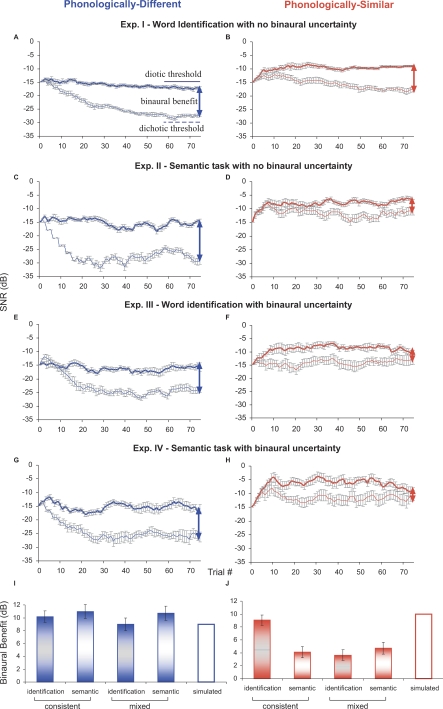
Results of Study 1, Experiments I–IV Left: results using phonologically different word pairs (blue). Right: results using phonologically similar pairs (red). (A–H) The dynamics of the adaptive threshold assessment as a function of trial number (averaged across subjects ± SEM, *n* = 10 for each of the eight conditions). The level of the signal was modified in relation to subject's performance (following a three down–one up adaptive procedure). Illustrations of diotic (thick curves) and dichotic (thin curves) thresholds, which are calculated as the mean of last five reversals (see Materials and Methods), are marked by dashed lines in (A). Thresholds are denoted in decibel SNR. Binaural benefits (vertical arrows in all panels) are calculated as the difference between the diotic and dichotic thresholds. (A and B) Experiment I: the identification task with no binaural uncertainty (consistent binaural protocol). (C and D) Experiment II: the semantic task with no binaural uncertainty (consistent binaural protocol. (E and F) Experiment III: the identification task with binaural uncertainty (mixed binaural protocol). (G and H) Experiment IV: the semantic task with binaural uncertainty (mixed binaural protocol). (I and J) A summary of the average binaural benefits obtained in Experiments I–IV (filled shaded bars), and the benefits calculated by an ideal listener model (open bars; see Figure S2 and Text S1), for phonologically different (left, [I]) and phonologically similar (right, [J]) pairs.

Figure 1 (A and B) shows the average changes of signal level during the adaptive tracks in the diotic (thick lines) and dichotic (thin lines) blocks, for both types of word sets, respectively. The plots denote the estimated signal to noise ratio (SNR—the difference between stimulus and noise levels) in decibels, as a function of trial number during the assessment. The initial SNR reflects an experimenter-selected level, but subsequent signal levels were set according to performance. Typically, a steady state level of performance is reached by the 40th trial, reflecting the SNR needed to attain 80% correct (which we use here as the discrimination threshold).

As expected, the diotic threshold for discriminating between phonologically similar words (Figure 1B, thick red curve) was higher than that for discriminating between phonologically different words (Figure 1A, thick blue curve), since this discrimination is more difficult. However, binaural benefits for both types of word pairs were similar, and reached 9–10 dB (10.2 ± 0.9 dB and 9.1 ± 0.8 dB for phonologically different and phonologically similar sets, respectively; *F*(1,18) = 0.84, not significant [n.s.]). In both cases, the measured binaural benefits reached the benefits predicted by the ideal listener calculations (see Figure S2 for full details). The large binaural benefit obtained with the phonologically similar words might seem surprising given their perceived similarity. However, as shown by the ideal listener calculations, low-level representations of the phonologically similar pair are distinct enough and contain informative binaural cues (Figure S1).

These results show that, in line with previous reports, under these simple conditions, binaural benefits reach ideal listener levels both when discriminating between phonologically similar words and when discriminating between phonologically different word pairs. These results are therefore consistent with all three views (Table 1, Experiment I).

### Experiment II—Semantic Task with No Binaural Uncertainty

In Experiment II, we manipulated the nature of the behavioral task without modifying its absolute level of difficulty. Semantic processing was not necessary in Experiment I, in which listeners were asked only to discriminate between the two words. Thus, in Experiment I, listeners could have used any low-level acoustic cue that differentiated between the two stimuli. We now wanted to ensure that listeners would process word meaning, as they typically do in more ecological conditions. In Experiment II, we therefore used a semantic-association task in which participants were asked to determine whether a visually presented word is semantically related to the auditory word, which was chosen from the same two-word set used in Experiment I. Visual presentation was brief, and subjects were instructed to respond immediately after stimulus presentation, imposing temporal constraints on the behavioral task (see below). The visually presented word in each trial was randomly selected from a large word set, inducing cross-trial variability in the association required and, hence, forcing semantic processing anew in every trial. Yet, low-level acoustic information was identical to that of Experiment I since the same two-word auditory sets were used, and the diotic and dichotic configurations were administered in separate blocks (consistent).

Introducing the semantic requirement did not affect task difficulty, as measured by absolute diotic thresholds, either for the phonologically different (−15.3 ± 0.8 dB for the semantic-association task; −16.9 ± 0.8 dB for the identification task; *F*(1,36) = 0.46, n.s.; compare Figure 1C and Figure 1A) or for the phonologically similar pair (−8.9 ± 0.5 dB for the semantic-association task; −9 ± 0.4 dB for the identification task; *F*(1,35) = 3.9, n.s.; compare Figure 1D and Figure 1B). However, its impact on binaural benefits greatly differed between these conditions. When the semantic task was performed with the phonologically different pair, binaural benefits remained as large as those of an ideal listener as measured in Experiment I (10.9 ± 1 dB compared with 10.2 ± 0.9 dB for the identification task; no effect of task: *F*(1,36) = 1.5, n.s.; Figure 1C and 1I). However, when the task was performed with the phonologically similar pair, dichotic thresholds were elevated, i.e., binaural benefits decreased (4.1 ± 0.9 dB compared with 9.1 ± 0.8 dB for the identification task; effect of task: *F*(1,36) = 5.3; *p* < 0.03; Figure 1D and 1J). The differences between performance with the phonologically similar and phonologically different sets cannot be attributed to differences in response times (RTs), as those were the same for the two word pairs used (672 ± 66 ms and 670 ± 112 ms for the phonologically similar and phonologically different pairs; *t*-test: *t* = −0.13, *df* = 17, n.s.).

The finding that binaural benefits remained equivalent to those of an ideal listener when the semantic task involved phonologically different words is in line with the unlimited view, which predicts full use of low-level information. However, this account cannot explain the failure of an ideal listener model to account for binaural benefits in the case of phonologically similar words. Since absolute diotic thresholds were not increased, there is no basis on which to assume an increase in perceptual or cognitive load. Moreover, had an increase in attentional load occurred with no impact on absolute thresholds, it should have reduced the ability to use binaural cues for both pair types. Thus, the ideal listener levels of binaural benefits for phonologically different words, but poorer benefits for phonologically similar words, are inconsistent with both the unlimited view and with the limited capacity view, but are in line with RHT predictions (Table 1, Experiment II).

### Experiment III—Word Identification with Binaural Uncertainty

In Experiment III, we asked whether introducing uncertainty in the low-level binaural configuration affects the use of binaural cues. We used the same word sets and the same identification task as in Experiment I. However, the diotic and dichotic configurations were randomly interleaved across trials (“mixed”). This manipulation therefore caused the low-level binaural cues required for correct performance to vary from trial to trial. Yet, the higher-level phonological and semantic representations as well as the definition of task demands were identical to those of Experiment I.

As expected, absolute diotic thresholds were not affected by this binaural variability, either for the phonologically similar pair (−9.8 ± 0.5 dB and −9 ± 0.4 dB in Experiments III and I, plotted in Figure 1F and 1B, respectively; effect of protocol: *F*(1,35) = 0.6, n.s.) or for the phonologically different pair (−16 ± 0.8 dB compared with −16.9 ± 0.8 dB, plotted in Figure 1E and 1A, respectively; effect of protocol: *F*(1,36) = 0.19, n.s.). However, the use of binaural cues for discriminating between phonologically similar words was substantially smaller than that predicted by the ideal listener model (3.6 ± 0.8 dB compared with 9.1 ± 0.8 dB for the consistent protocol; *F*(1,36) = 7.96, *p* < 0.01), whereas for the phonologically different words, binaural benefits remained equivalent to those of the ideal listener (9 ± 1 dB and 10.2 ± 0.9 dB in the mixed and consistent protocols, respectively; no effect of protocol: *F*(1,36) = 0.5, n.s. see Figure S2, right).

Thus, introducing variability of the informative low-level information across trials disabled listeners from reaching ideal listener levels of binaural benefits when discriminating between phonologically similar words, but not when discriminating between phonologically different words. The results of this experiment pose an even greater challenge to the limited capacity view, since not only measurable (diotic) thresholds remained the same, but also introspective task demands were exactly as in Experiment I (Table 1, Experiment III). In post-test questionnaires, listeners failed to report any information regarding the binaural configuration, indicating that they were not aware of this low-level variability.

### Experiment IV—Semantic Task with Binaural Uncertainty

In Experiment IV, we combined the two types of manipulations. Subjects were asked to perform a semantic-association task (similar to the one in Experiment II) while diotic and dichotic configurations were mixed (i.e., randomly interleaved) within the block (as in Experiment III).

The results of this experiment (Figure 1G and 1H) were similar to those of Experiments II and III. Thus, having the two constraints together yielded the same results that each of them produced separately. Absolute diotic thresholds were similar to those of Experiment I for both phonologically different pairs (−15.2 ± 1.2 dB and −16.9 ± 0.8 dB in Experiments IV and I, respectively; interaction of task × protocol: *F*(1,36) = 1.4, n.s.) and for phonologically similar pairs (−7.6 ± 0.7 dB and −9 ± 0.4 dB for Experiments IV and I, respectively; no significant interaction of task × protocol: *F*(1,35) = 2.9, n.s.). However, binaural benefits were similar to those of Experiments II and III. They matched those of the ideal listener (as measured for Experiment III) for the phonologically different word set (10.7 ± 1.1 dB; no significant interaction of task × protocol: *F*(1,36) = 0.21, n.s.), and were significantly poorer than the ideal listener prediction for the phonologically similar words (4.7 ± 0.9; significant interaction of task × protocol: *F*(1,36) = 12.7, *p* < 0.005; see Table I, Experiment IV). As in Experiment II, RTs were kept below 1 s, and did not differ significantly between phonologically different (722 ± 70 ms) and phonologically similar (723 ± 95 ms) pairs (*t*-test: *t* = −0.14, *df* = 17, n.s.).

### Summary and Discussion of Study 1

In Study 1, we found that, in line with the unlimited view, full use of binaural information can be obtained with both phonologically similar and phonologically different word sets. However, the unlimited view fails to predict binaural benefits for phonologically similar words when low-level cross-trial uncertainty is introduced. A similar drop in utilization of low-level information is found when semantic processing is required. These failures cannot be explained by limited capacity models either (e.g., [45,46,71]), since these manipulations did not increase task difficulty, as reflected by the unchanged diotic thresholds (Experiments II–IV), and were in some cases transparent to participants (Experiment III). Table 1 summarizes the predictions and results of the three views for Experiments I–IV.

In order to verify that this set of results systematically characterizes the manipulations we introduced and is not specific to the two word pairs that we used in Study 1, we fully replicated Study 1 with two other word pairs, and obtained similar results (detailed in Figure S5).

### Study 2—Manipulating Task Difficulty

In Study 1, we manipulated explicit (Experiment II) and implicit (Experiment III) task requirements without modifying task difficulty, and assessed the impact of these manipulations on binaural benefits. In Study 2, we designed manipulations that were aimed at modifying task difficulty (diotic thresholds) in order to assess whether this type of change affects the use of binaural cues, as predicted by the limited capacity view.

### Experiment I—Manipulating Set Size (“Cognitive Load”)

In this experiment, we increased the cognitive load of the task by increasing stimulus set size. This manipulation (increasing “memory set size”) has been shown to increase the cognitive load both in the visual (e.g., [76]) and in the auditory (e.g., [77]) domains. We expected that diotic thresholds would increase and tested the resulting effects on binaural benefits. In the new condition with high cognitive load, the presented word on a given trial was selected from a set of ten words rather than two words. Sets were composed of either phonologically different (ten *different* words) or phonologically similar (five pairs of *similar* words) words. We used the mixed binaural protocol with randomly interleaved diotic and dichotic trials (as was used in Experiments III and IV of Study 1).

**Figure 2:**
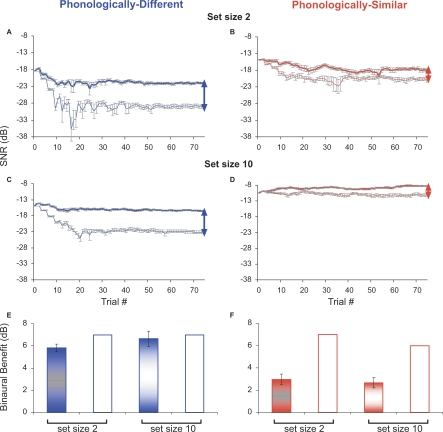
Results of Study 2, Experiments I Left: results using phonologically different words (blue). Right: results using phonologically similar words (red). (A–D) The dynamics of the adaptive threshold assessment as a function of trial number (averaged across subjects ± SEM, *n* = 25). Notations as in Figure 1. Vertical arrows denote binaural benefits. All measurements were done using the mixed binaural protocol. (A and B) An identification task using a set size of two words. (C and D) An identification task using a set size of ten words. (E and F) A summary of the average binaural benefits obtained in the experiment (filled shaded bars) and the benefits calculated by the ideal listener model (open bars; see Figure S3), for the set size 2 and set size 10 conditions.

As expected, increasing the set size from two to ten significantly increased diotic thresholds for both the phonologically different set (from −21.8 ± 0.6 dB to −16.7 ± 0.2 dB SNR; Figure 2A vs. 2C) and the phonologically similar set (from −18 ± 1.1 dB to −8 ± 0.2 dB SNR; Figure 2B vs. 2D; *F*(1,18) = 391, *p* < 0.00001). A larger increase in thresholds was found for the phonologically similar set (a significant interaction of set size × similarity: *F*(1,18) = 71.8, *p* < 0.00001). However, binaural benefits did not significantly change (no effect of set size *F*(1,18) = 0.09, n.s.). They were quite large for the phonologically different words (5.8 ± 0.3 dB and 6.6 ± 0.7 dB for set sizes of ten and two, respectively, Figure 2E), matching those of the ideal listener (Figure 2E, see details in Figure S3). They were smaller for the phonologically similar words (3 ± 0.5 dB and 2.3 ± 0.3 dB for set sizes of ten and two, respectively), and did not reach the values predicted by the ideal listener model (Figures 2F and S3). Thus, binaural benefits reached ideal listener levels for phonologically different words, but failed to reach these levels for phonologically similar words, regardless of set size (a significant effect of phonological similarity, *F*(1,18) = 51, *p* < 0.0001; no significant interaction of set size × similarity, *F*(1,18) = 3.7, n.s.).

The results of this experiment show that although increasing the cognitive load (by increasing the set size from two to ten) yields the expected increase in diotic identification thresholds, it does not change binaural benefits. This experiment thus clearly dissociates between task difficulty and the ability to use low-level information, and its results are therefore inconsistent with limited capacity models, but are in line with RHT predictions (Table 2, Experiment I).

### Experiment II—Manipulating Success Level (“Perceptual Load”)

In this experiment, we increased the perceptual load by modifying the adaptive procedure to a procedure that converges at approximately 60% rather than 80% correct [78]. Subjects reported that this protocol “felt more difficult,” presumably due to the lower SNRs at which most stimulus presentations occurred. We asked whether this change in difficulty affects binaural benefits. We calculated ideal listener performance for both levels of difficulty and compared them to the measured binaural benefits.

**Figure 3:**
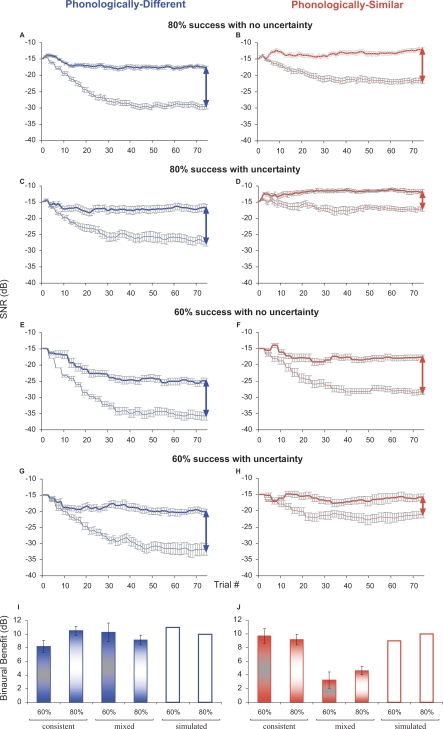
Results of Study 2, Experiments II Left: results using phonologically different word pairs (blue). Right: results using phonologically similar pairs (red). (A–H) The dynamics of the adaptive threshold assessment for identification of word pairs as a function of trial number (averaged across subjects ± SEM, *n* = 15). Notations as in Figure 1. Vertical arrows denote binaural benefits. (A and B) The adaptive protocol converging to 80% correct identification with no uncertainty (i.e., using the consistent binaural protocol). (C and D) The adaptive protocol converging to 80% correct identification with uncertainty (mixed binaural protocol). (E and F) The adaptive protocol converging to 60% correct identification with no uncertainty (consistent binaural protocol). (G and H) The adaptive protocol converging to 60% correct identification with uncertainty (mixed binaural protocol). (I and J) A summary of the average binaural benefits obtained in the experiment (filled shaded bars), and the benefits calculated by an ideal listener model (open bars; see Figure S4).

First, we replicated Experiments I and III of Study 1, using the original adaptive procedure converging at 80% correct, using other word pairs (/barul/ vs. /parul/ and /dilen/ vs. /talug/, respectively). Indeed, when the task required identification and was administered with the consistent binaural protocol (with separate measurements of the diotic and dichotic thresholds, as in Study 1, Experiment I), binaural benefits reached the ideal listener levels, of 9–10 dB, for both word sets (10.5 ± 0.7 dB and 9.2 ± 0.8 dB for the phonologically different and phonologically similar pairs, respectively; Figure 3A and 3B). However, only the phonologically different set yielded similar benefits under the mixed binaural protocol, when diotic and dichotic trials were randomly interleaved (9.2 ± 0.7 dB compared with 4.6 ± 0.6 dB obtained with the phonologically similar pair; Figure 3C and 3D), fully replicating Experiments I and III of Study 1 (see Table 2, Experiment II).

We then asked whether a similar pattern of binaural benefits would be found with the adaptive protocol converging to approximately 60% success in the task, rather than to 80% success. As expected, diotic thresholds for both phonologically similar and phonologically different pairs were lower for the 60% correct condition (Figure 3E–3H) compared with the 80% correct condition (analysis of variance [ANOVA]: percent correct: *F*(1,23) = 52.6, *p* < 0.00001; similarity: *F*(1,23) = 18.2; *p* < 0.0005; and between-subjects factor of binaural protocol: *F*(1,23) = 1.9, n.s.). Moreover, binaural benefits obtained with 60% correct had the same pattern, and did not significantly differ from those obtained with 80% correct (*F*(1,23) = 0.43, n.s.). They were large for both sets under the consistent binaural protocol (7.8 ± 0.8 dB and 9.7 ± 1 dB for phonologically different and phonologically similar pairs, respectively). Yet, only the phonologically different set yielded similar binaural benefits with the mixed binaural protocol (10.5 ± 1.5 compared with 3.2 ± 1.2 obtained with the phonologically similar pair). Thus, there was a significant effect of protocol (*F*(1,23) = 8.9, *p* < 0.008) and a significant interaction between similarity and protocol (*F*(1,23) = 14.9, *p* < 0.001).

We calculated the ideal listener performance for these conditions as well. Discrimination between phonologically similar words under the consistent binaural protocol at 80% correct was used for calculating the variances in neural activity (these were the values used at all other ideal listener calculations in this paper). We then calculated ideal listener performance for all other conditions. The ideal listener model accounted for performance in all conditions when discriminating between phonologically different words pairs, but only for the performance in the consistent binaural protocol when discriminating between phonologically similar words (see Figure S4).

### Summary of Study 2

The two experiments of Study 2 show different manipulations that affect task difficulty and yet do not affect the use of binaural cues. The finding that increased difficulty does not decrease the use of low-level information indicates that, in contrast to the limited capacity view, attentional load is not the bottleneck for our ability to use low-level information. Table 2 summarizes the predictions and results of the three views for Experiments I and II.

## Discussion

We tested the use of low-level information for the extraction of speech from noise, and contrasted the predictions of three theoretical frames, as listed in Tables 1 and 2. We found that when the set of stimuli was composed of phonologically different words, binaural benefits matched those predicted by the ideal listener model under different types of task requirements (Study 1, Experiments I and II), different levels of task difficulty (Study 2), and different binaural protocols (Study 1, Experiments III and IV). Thus, they were fully accounted for by the unlimited view, which predicts ideal listener levels of utilization under all conditions. However, when exactly the same conditions were administered with phonologically similar pairs, binaural benefits were substantially lower than those predicted by the ideal listener model under most conditions. This difference cannot be explained in terms of differences in available low-level binaural information, since the ideal listener model explicitly accounts for these differences (see Text S1). Moreover, the binaural benefits predicted by the ideal listener model (and hence by the unlimited view) were achieved in Experiment I of Study 1 and in Experiment II of Study 2. Thus, contrary to the unlimited view (which was supported by, e.g., [32,33,38,39,46]), low-level information is not always fully used. The results of Study 2 further dissociate between task difficulty and the ability to use binaural cues, thus ruling out limited-capacity models of attention by which performance is expected to be limited by task difficulty per se (e.g., [65,66,79,80]).

We conclude that there are indeed constraints on the use of low-level information, but these constraints have to be formulated in terms of the properties of the stimulus set rather than in terms of behavioral difficulty, or general cognitive or attentional demands. The main difference between the two types of stimulus sets that we used is the phonological contrast between the words composing them. The acoustic contrast was large for both types of sets, and hence, at low representation levels both word sets presumably had distinct, nonoverlapping representations (see Figure S1 and Text S1). However, the phonological contrast was small for one type and large for the other. Hence, for the similar sets, high-level phonological representations of the words were close and largely overlapping, whereas for the different sets they were distant. We therefore conclude that the main factor determining whether the use of low-level information would reach ideal listener levels is the nature of high-level representations of the stimuli composing the stimulus set. Among the relevant theoretical accounts, only the RHT [69,81,82] concretely addresses the relations between the use of low-level information for perception and the underlying hierarchy of representations. Though RHT was originally derived to explain visual perception, we argue here that it also applies to the auditory system.

### Binaural Benefits and the Reverse Hierarchy Theory

The basic tenets of RHT are the presence of a local-to-global hierarchy of stimulus representations, and the presence of massive feedback connections throughout this hierarchy. Feedback connections are well established throughout the brain [83–85]. There is also an increasing amount of evidence for an auditory processing hierarchy in which lower stations represent acoustic features of sounds, whereas higher stations represent sounds more abstractly [1–8]. Along this hierarchy, acoustic fidelity is presumably gradually replaced by ecologically relevant representations [17–21]. In analogy to the visual system, low-level representations are determined by the physical (acoustic or visual) nature of the stimulus, and high-level representations converge across different low-level representations that denote the same objects or events.

Anatomically, the lower, acoustic levels may roughly correspond to the stages up to, and including, the inferior colliculus (IC; e.g., [19]), whereas the more abstract levels, though less well understood, may correspond to cortical areas. For example, according to some recent imaging data, cortical areas ventral (“belt”) and posterior (“parabelt”) to A1, and portions of the superior temporal sulcus (STS), process temporal and spectral feature combinations that may be related to phoneme discrimination [23–28]. Cortical areas in posterior middle temporal regions [23,24,29–31,86] may process semantic information.

RHT asserts that perception is based, by default, on stimulus representations at higher levels of the processing hierarchy, which are immediately accessible to perception. This functional structure allows rapid, and yet crude, evaluation of meaningful objects and events. The finding that binaural benefits utilize all low-level information in the case of phonologically different words is therefore consistent with RHT assertions. This is because the phonological representations of phonologically distant stimuli are as informative as the low-level representations, and therefore, can be used to achieve the performance level suggested by ideal observer models.

However, in the case of phonologically similar words, the phonological representations of the two words are close and largely overlapping, resulting in information loss about the acoustic differences between them, since much of the acoustic difference between the two words is irrelevant at the phonological level and is therefore not explicitly represented (e.g., [87]; see Figure S1). To discriminate between the two words, it is necessary to access the discriminative features that are represented at lower, acoustic representation levels. These features depend on the binaural configuration [88,89]; they are energy cues in diotic trials and correlation cues in dichotic trials, which are presumably coded in different low-level representations [88,90]. According to RHT, access to the appropriate lower level representations requires a backward search down the auditory hierarchy, since there are a number of possibly informative low-level representations. For example, there are monaural pathways through the ventral and dorsal cochlear nuclei, binaural pathways through the medial and lateral superior olivary nuclei (SOC), and pathways through the nuclei of the lateral lemniscus, all of which reach the inferior colliculus and remain partially segregated there (see [90]). RHT postulates that the backward search for the specific low-level neural population that best represents the discriminative acoustic features is difficult. In particular, it is gradual, and *cannot* be conducted on a trial-by-trial basis; RHT suggests that this search is aimed at allocating a population that is consistently informative across several trials [70].

This logic therefore accounts for the substantially reduced binaural benefits in the case of phonologically similar pairs when binaural conditions vary in an uncertain (mixed) manner across trials: this presumably requires access to different low-level populations that vary from trial to trial. Given that identification of the most discriminative population requires several stimulus repetitions, a successful backward search can be achieved only in the consistent protocol.

Listeners' limited ability to use binaural information in the semantic-association task, even when the binaural configuration is consistent across trials, can also be accounted for by this logic. Comprehending the visually presented word, which immediately follows the auditory presentation and changes on a trial-by-trial basis, requires access to higher, semantic representation levels on every trial and interferes with the backward search for informative low-level representations. Therefore, the requirement for semantic processing prevents access to low-level representations, and thus limits the use of binaural information in the case of phonologically similar words.

The same RHT-based interpretation can also explain many examples from previous studies showing a tradeoff between understanding speech, i.e., processing its semantic content (based on high-level representations), and perceiving its fine details, when the latter requires direct access to appropriate low-level populations [91–96]. Similarly, auditory attention (particularly “informational masking”) studies report impaired use of low-level information when high-level confusion between the target and the masker is introduced (e.g., [58,59,62]). According to RHT, the low-level degree of segregation between the target and the masker could have been obtained had a gradual backward search for the informative low-level representation been applied successfully. However, in these studies, target selection is based on high-level representations (e.g., target is defined by its semantic content [59,60,62]). Accessing these high-level representations disables a concurrent backward search. Thus, according to RHT, similar constraints underlie the limited use of low-level information in these studies and in our semantic-association task using phonologically similar word pairs.

### Reverse Hierarchy Theory, Speech Perception, and Ecology

We propose that the immediate access to higher levels of the processing hierarchy allows fast word identification in an overall slow system [20]. Specifically, in general conversational situations, the context usually provides prior information that limits the expected word set to words that are semantically related, but are typically phonologically dissimilar. We now find that in these situations, the auditory system discriminates as well as an ideal listener regardless of the attentional load imposed by the conversation. Thus, in the majority of the daily discriminations we need, the system fully utilizes all relevant information. However, in those cases that require finer phonological discriminations, and ideal listener levels cannot be provided by the broad, abstract high-level representations, a different process occurs. Thus, for example, when the speaker might say either /day/ or /bay/ outside of context, we are likely to ask “what?”, “forcing” the speaker to repeat, perhaps at a higher signal level, which improves SNR. In parallel, an implicit attempt to apply a backward search to find more discriminative low-level representations is made. A successful backward search requires a relatively specific expectation (/day/ vs. /bay/), another repetition of the same condition, and disables concurrent semantic processing. Yet, it can provide a better discrimination, even under the less common conditions that require such access.

Taken together, the auditory system seems to favor ecologically more likely conditions and yet retains flexibility for the less likely ones. Discriminations that are prevalent in natural situations are fast and still use all low-level information, whereas discriminations that are less likely to occur are either fast or use all low-level information. The results presented here, however, show that the auditory system cannot achieve both. These results are in line with our earlier results in the visual system [69,70], which showed that it too can attain low-level accuracy only under similarly limited conditions and at the cost of concurrently broad object perception. This resemblance suggests that similar defaults and tradeoffs characterize the relations between processing hierarchies and perception at the various sensory modalities.

## Materials and Methods

### Behavioral experiments—Participants.

In Study 1, we tested a total of 80 subjects, whose mean age was 24 ± 3 y. In each of the four experiments (I–IV), we tested 20 subjects, ten in each type of phonological similarity (phonologically different and phonologically similar). Thus, different subjects were tested in the different experiments and different conditions, to avoid effects of task and protocol learning. In Study 2, we tested a total of 40 subjects (mean age: 24 ± 3 y): 25 subjects in Experiment I and 15 subjects in Experiment II. In this study, each subject performed all conditions in each experiment. All subjects were undergraduate students at the Hebrew University of Jerusalem. All were native Hebrew speakers, had normal hearing, and gave their informed consent for participation.

### Behavioral experiments—Stimuli*.*

Stimuli were either disyllabic pseudowords (Experiment II of Study 2) or familiar Hebrew words, all recorded by the same female speaker. Each word had two different instances. Overall root mean square (RMS) and duration were equated for all words. In Study 1, the same word pairs were used in all four experiments: a phonologically similar pair, within which the difference was in a single phoneme (/tamid/ vs. /amid/), and a phonologically different pair, in which words differed in most phonemes (/tamid/ vs. /chalom/). In Experiment I of Study 2, we used the following ten-word sets: a set of ten Hebrew digits for the phonologically different condition (/efes/, /ahat/, /shtaim/, /shalosh/, /arba/, /hamesh/, /shesh/, /sheva/, /shmone/, and /tesha/), and a set of ten familiar words, composed of five phonologically similar pairs, for the phonologically similar condition (/shalom/ vs. /chalom/, /tamid/ vs. /amid/, /banuy/ vs. /panuy/, /tmuna/ vs. /tluna/, and /shanim/ vs. /panim/). For the “set size 2” condition, we used one set of digits (4 /arba/ and 9 /tesha/) for the phonologically different condition, and a pair of similar words (/shalom/ vs. /chalom/) out of the list of ten words for the phonologically similar condition. In Experiment II of Study 2, we used pairs of phonologically similar (/barul/ vs. /parul/) and phonologically different (/dilen/ vs. /talug/) pseudowords.

The masking noise in both studies was speech noise [75], played at a constant level of 66 dB SPL (sound pressure level) to both ears. The noise was always identical in both ears. Words were played in two different configurations: diotic (*N_0_S_0_*), in which the word was added to the noise in-phase at both ears, and dichotic (*N_0_S_π_*), in which the word was phase-inverted in one of the ears before it was added to the noise. The duration of the noise was 1.4 s, whereas the duration of the word was 0.8 s. Thus, the noise began 0.3 s before and ended 0.3 s after the word. All stimuli were digitally played by a TDT system III signal generator (Tucker Davis Technologies), and presented to listeners through HD-256 Sennheiser headphones.

### Behavioral experiments—Procedure.

All experiments were conducted in a sound-attenuated room.

*Study 1—Experiments I and III* (*identification*). In each trial, one of two possible words was presented, masked by noise, and the listener had to press the left/right button on the computer screen whose label matched the played word. Feedback was given after every button press: a positive feedback for correct responses (happy face) and a negative feedback for incorrect responses (sad face).

*Study 1—Experiments II and IV* (*semantic-association*)*.* In each trial, one of the two words was presented in noise. Immediately following the auditory presentation, a word was visually presented on the screen for 500 ms. Listeners had to decide whether the acoustically presented word was semantically related to the visually presented word. In each trial, the visually presented word was selected from a set of 20 different words, ten of which were semantically associated to one auditory word and ten to the other word. Subjects had to press the right button (green: “match”) if it matched the auditory word and the left button (red: “no match”) if it did not. Feedback protocol was the same as for the identification experiments. Subjects performing these experiments were given a short, 20-trial training session prior to the experiment. Subjects were instructed to respond accurately and quickly. We verified that they did so by measuring their RTs (from the end of the visual presentation until button press). Average RTs were calculated for the 75 trials comprising each assessment, and were further averaged across the diotic and dichotic binaural configurations. Comparison of RTs between the relevant word pairs was performed using nonpaired two-tailed Student *t*-tests.

*Study 2—Experiment I* (*cognitive load*)*.* For the “set size 2” condition, identification thresholds were measured similarly to those measured in Experiments I and III of Study 1. For the “set size 10” condition, subjects heard on each trial one of the ten words, masked in noise, and were requested to report the word to the experimenter. The experimenter pressed a green or red button following a correct or incorrect response, respectively. For this condition, subjects were first given a short practice of 20 trials in which they had to correctly identify the words presented without any masking noise.

*Study 2—Experiment II* (*perceptual load*). Identification thresholds were measured similarly to those measured in Experiments I and III of Study 1 (see above).

*Protocol for measuring thresholds.* Thresholds for correct identification were measured in both studies using an adaptive staircase procedure [78]. In most experiments (excluding part of Experiment II of Study 2), thresholds were measured using a three down–one up adaptive staircase procedure, converging at 79.4% correct. In Experiment II of Study 2, the “60% correct” condition was measured using another up–down procedure, converging to 61.8% correct. In this method, signal level was decreased after at least two consecutive successes out of every three trials. Signal level was increased after any of the other five combinations of successes and errors out of every three trials.

The level of the masking noise was kept constant while the presentation level of the word was adaptively varied (see left panel of Figure 1A). In all experiments, we used five different step sizes, beginning at 2 dB and switching to smaller steps after every four reversals (1, 0.5, 0.2, and 0.1 dB). Each experiment was composed of 75 trials for each binaural configuration. Thresholds were calculated as the arithmetic mean of signal amplitude in the last five reversals. The binaural benefit was calculated as the difference (in decibels) between the measured diotic and dichotic thresholds (illustrated in Figure 1A). In Study 1, each subject was administered one assessment per word pair with each binaural configuration (i.e., 150 trials with each word pair). Each subject performed the same experiment twice, with two different word pairs. Both were either phonologically similar or phonologically different. In Study 2, each subject performed all conditions of each experiment (different subjects for Experiments I and II). Thus, in Experiment I of Study 2, each subject performed both set size 2 and 10 conditions, with both phonologically different and phonologically similar pairs. In Experiment II of Study 2, each subject performed both the 60% and 80% correct conditions, with both types of pseudoword pairs.

*Binaural protocol.* In Study 1, two groups of subjects (Experiments I and II) performed the experiments with a consistent binaural protocol. In these groups, diotic and dichotic configurations were measured in different experimental blocks of 75 trials each, administered in immediate succession. The order of the sessions was counterbalanced between subjects. The other two groups (Experiments III and IV) performed the task with a mixed binaural protocol. In this protocol, diotic and dichotic configurations were randomly interleaved across the block: on each trial, either a diotic or a dichotic configuration was chosen uniformly at random. The interleaved blocks consisted of 150 trials, 75 per each binaural configuration. Although the configurations were administered in an interleaved manner, the adaptive thresholds were tracked separately throughout the assessment. In Study 2, Experiment I was administered using only the mixed binaural protocol, whereas Experiment II was administered using both protocols.

### Data analysis

*Study 1.* We used univariate analysis with between-subject factors of task (two levels: identification and semantic-association) and protocol (two levels: consistent and mixed), thus comparing results of Experiments I–IV. Binaural benefits and diotic thresholds were separately used as the dependent variables. Data analysis was performed separately for each word set (phonologically similar and phonologically different sets). Comparison of RTs between the relevant word pairs was performed using nonpaired two-tailed Student *t*-tests.

*Study 2.* In Experiment 1, we used ANOVA with within-subject factors of set size (two levels: 2 and 10) and similarity (two levels: phonologically similar and phonologically different). In Experiment 2, we used ANOVA with within-subject factors of percent correct (two levels: 60% and 80% correct) and similarity (two levels: similar and different), and a between-subject factor of protocol (two levels: consistent and mixed). Results were corrected using the Greenhouse-Geisser correction.

### “Ideal listener” simulation.

We used an ideal listener model to calculate performance given access to all low-level information. The model consisted of a peripheral stage ending with a binaural cross-correlator (roughly simulating the auditory system up to the level of the SOC), followed by an ideal listener under the assumption of additive Gaussian noise. The stimuli used in the behavioral experiments were filtered into narrow frequency bands, half-wave rectified, compressed, and low-pass filtered at 1,200 Hz, generating a simulated activity pattern of auditory nerve fibers (using the AIM software package [97]; 32 bands equally spaced along the basilar membrane between 100 and 4,000 Hz). The signals in each of these bands were used to calculate energy and binaural correlation signals, sampled every 10 ms. Close to threshold, the binaural correlation is dominated by the in-phase noise, and is maximal at an interaural delay of zero. Therefore, only this delay was used. The energy and correlation signals (as a function of frequency and time) were fed to an optimal decision maker, which compared them to stored templates of each of the possible words. Under the assumption of Gaussian noise, optimal decision consisted of selecting the template that was closer (in the least squared difference sense) to the incoming signal. Dynamic time warping (DTW) was used for computing the distance between the input signals and the stored templates, simulating templates with various temporal relations between their subparts. Consequently, the optimal decision maker had full access to the low-level pattern of activation on the one hand, and to temporally flexible representations of the stimulus set on the other hand.

The Gaussian noise was assumed to be identically distributed and independent in each frequency and time bin, and its variance was determined by fitting the diotic and dichotic thresholds of a single word pair (/barul/ and /parul/) in a single condition: consistent binaural protocol with an adaptive three down–one up procedure converging to 80% correct identification. These words were used as the phonologically similar pair in Study 2, Experiment II. The estimated thresholds for Study 1 (all experiments), Study 2 Experiment I, and Study 2 Experiment II (phonologically different word pair and all other conditions for the phonologically similar pair) and the replication reported in Figure S5 were all computed with these estimates for the variances. Thus, in all other cases (except for /barul/ and /parul/ under the consistent binaural protocol), the simulation had no free parameters. Detailed description of the simulation can be found in Text S1 online.

## Supporting Information

Figure S1 Methods (A and B) The auditory nerve activity patterns for the left and right ears for the phonologically similar pseudowords /barul/ (A) and /parul/ (B) at a SNR of +10 dB. Patterns are calculated at 32 frequency channels between 100–4,000 Hz, at 80 time bins of 10 ms each. Note the difference in patterns, despite the similarity of the words.
(C and D) The energy (left of each panel) and binaural correlation (right of each panel) templates for the same pseudowords, calculated from the auditory nerve pattern in panels (A) and (B).
(E) Euclidean (left) and DTW (right) distances calculated for a pair of phonologically different (blue bars) and a pair of phonologically similar (red bars) words. Distances are normalized by standard deviations. Euclidean distances are essentially equal for both pairs, whereas the DTW distance is much smaller for the phonologically similar pair (see text). The use of the DTW distance was needed in order to account for the higher discrimination thresholds of phonologically similar word pairs.
(768 KB TIF)

Figure S2 Comparing Results of Ideal Listener Model to Experimental Results of Study 1 Graphs compare simulated (empty bars) and experimental (filled bars) thresholds (A–D) and binaural benefits (E and F) for both the phonologically different (/tamid/ vs. /chalom/; blue bars) and phonologically similar (/tamid/ vs. /amid/; red bars) word pairs, under both consistent (left; Experiment I) and mixed (right; Experiment III) binaural protocols.
(A and B) Diotic thresholds; (C and D) dichotic thresholds; (E and F) binaural benefits. Note the difference between simulated and experimental binaural benefits for the phonologically similar words, measured under the mixed binaural protocol (red bars of [F]).
(2.11 MB EPS)

Figure S3 Comparing Results of Ideal Listener Model to Experimental Results of Experiment I of Study 2 Results are compared for set sizes of two (left) and ten (right) of phonologically different and phonologically similar word pairs. Notations as in Figure S2. (A and B) Diotic thresholds; (C and D) dichotic thresholds; (E and F) binaural benefits. Note the difference between simulated and measured binaural benefits for the phonologically similar pair (red bars).
(2.15 MB EPS)

Figure S4 Comparing Results of Ideal Listener Model to Experimental Results of Experiment II of Study 2 Results are compared for performance levels of 60% and 80% correct for both phonologically different (blue bars) and phonologically similar (red bars) pseudoword pairs, measured under consistent (left) and mixed (right) binaural protocols. Notations as in Figure S2. (A and B) Diotic thresholds; (C and D) dichotic thresholds; (E and F) binaural benefits.
(3.11 MB EPS)

Figure S5 Replication of the Results of Study 1, Experiments I–IV, with Two Other Pairs of Words (/Sikum/ versus /Amid/ and /Shalom/ versus /Chalom/) Left: the phonologically different word pair (blue). Right: the phonologically similar pair (red). (A–D) Diotic and dichotic thresholds measured for Experiments I–IV of Study 1 (averaged across subjects ± standard error of the mean [SEM], *n* = 10 for each of the eight conditions). Binaural benefits are the differences between the two thresholds.
(A and B) Identification task with no binaural uncertainty (consistent protocol; left, Experiment I) and with binaural uncertainty (mixed protocol; right, Experiment III). Diotic thresholds were similar under both protocols (phonologically different: −25 ± 1.1 dB vs. −25 ± 0.7 dB under consistent and mixed binaural protocols, respectively; effect of protocol: *F*(1,35) = 0.07, n.s.; phonologically similar: −17.7 ± 0.5 dB vs. −18.6 ± 1.2 dB under consistent and mixed binaural protocols, respectively; *F*(1,35) = 0.16, n.s.). Binaural benefits were relatively large for both pairs under the consistent protocol (phonologically different: 7.2 ± 1.5 dB; phonologically similar: 5.2 ± 0.5 dB). Note that although the binaural benefit was a bit smaller for the phonologically similar pair, it still matched that simulated by the ideal listener model (see below and [E and F]). However, under the mixed binaural protocol, binaural benefit was reduced for the phonologically similar pair (1.9 ± 0.3 dB; *F*(1,35) = 16.8; *p* < 0.0005), but remained at the ideal listener level for the phonologically different pair (7.7 ± 0.5 dB, *F*(1,35) = 0.17, n.s.).
(C and D) Semantic-association task with no uncertainty (consistent protocol; left, Experiment II) and with uncertainty (mixed protocol; right, Experiment IV). Diotic thresholds were similar to those measured in the identification task (compare to [A and B]) for both phonologically different (−23 ± 1 dB and −23 ± 0.8 dB in the consistent and mixed binaural protocols; [C]) and phonologically similar (−17.9 ± 0.8 dB and −16.3 ± 0.6 dB; no effect of task: *F*(1,35) = 1.5, n.s.) pairs. Statistically, there was no significant interaction of task × protocol for both the phonologically similar (*F*(1,35) = 2.1, n.s.) and phonologically different (*F*(1,35) = 0.99, n.s.) pairs. Binaural benefits remained ideal-like only for the phonologically different pair (9.6 ± 0.7 dB and 8.2 ± 0.9 dB for the consistent and mixed binaural protocols; no effect of task: *F*(1,35) = 2, n.s.; no significant interaction of task × protocol: *F*(1,35) = 0.9, n.s.). However, for the phonologically similar pair, binaural benefits decreased (2.8 ± 0.4 dB and 2.6 ± 0.4 dB for consistent and mixed protocols, respectively; effect of task: *F*(1,35) = 4.4; *p* < 0.05; interaction: *F*(1,35) = 14.4, *p* < 0.001).
(E and F) A summary of the average binaural benefits (the difference between diotic and dichotic bars in each panel) obtained in Experiments I–IV (filled shaded bars), and the benefits calculated by an ideal listener model (open bars).
(2.13 MB EPS)

Text S1 Supplemental Methods and Results Description of the “ideal listener” simulation and its results on the various word stimuli used in Study 1 and 2.
(64 KB DOC)

## Abbreviations

DTW: dynamic time warping
n.s.: not significant
RHT: Reverse Hierarchy Theory
RT: response time
SNR: signal to noise ratio
SOC: superior olivary complex
