# Supplementary material for: Low-Level Information and High-Level Perception: The Case of Speech in Noise
Source: PLoS Biol. 2008 May 20;6(5):e126. doi: 10.1371/journal.pbio.0060126 (PMC2386842; doi:10.1371/journal.pbio.0060126)
Supplement: Supplementary file 1 [file pbio.0060126.sd001.doc]

**SUPPORTING INFORMATION**

### Ideal Listener Simulation

### Methods

1. ***Creating the templates***

*Simulating auditory nerve fibers*

The inputs consisted of the waveforms of the words used in our experiments (e.g. /barul/), masked by frozen speech noise of different levels (see **Results** in the main paper). The first step was to simulate the representations of the inputs at low levels of the auditory processing hierarchy. Due to peripheral processing, the neuronal representations at this stage have less information than the raw acoustic signals; some modeling of the peripheral processing is therefore necessary in order to correctly fit human performance [e.g. 1]. For each word, we simulated two configurations, diotic (N_0_S_0_) and dichotic (N_0_S_π_). In the diotic configuration, the inputs to the left and right ears were identical. In the dichotic configuration, the noise was the same for both ears, but the signal was inverted in the right ear before adding it to the noise. The activation of the peripheral frequency channels was calculated using the AIM software package [2], and included filtering, half-wave rectification, compression, and a low-pass filter with a corner frequency of 1200 Hz. In order to control for the effects of low-pass filtering on binaural benefits, we repeated the simulations with a low-pass filter at 500 Hz following half-wave rectification (as suggested by e.g. [3]). Since the results using the 500 Hz cutoff frequency were not substantially different from those using 1200 Hz as the cutoff frequency, they are not reported here. We simulated the activation of 32 channels in the frequency range of 100-4000 Hz for 800 ms (the standardized duration of each word). The auditory nerve activity patterns for the left and right ears are displayed in Figures S1a & S1b for two phonologically-similar pseudo-words /barul/ (Figure S1a) and /parul/ (Figure S1b), respectively, at a signal to noise ratio of +10dB. These words were used in Experiment II of Study 2. For each word, two patterns are presented: one for the left ear and one for the right ear. The two patterns for the two ears for each word are very similar, except for a phase shift in the fine structure of the activity at the low frequency channels (which cannot be seen at the resolution of the figure). The rapid fluctuations of the activation levels in the low-frequency channels are due to the pitch of the voiced segments in these words. Since the noise was frozen in the experiment, we created an activity pattern for each SNR level used in the experiment. We denote the left and right activity patterns before averaging as ANF_L_(f,t) and ANF_R_(f,t), respectively, with implicit dependence on the noise level and on the binaural configuration.

The activity patterns were used to generate ‘energy’ and ‘binaural correlation’ templates for each token and each SNR level. The absolute values of the simulated responses were smoothed by averaging over consecutive 10 ms periods, resulting in 80 time samples for each channel. The energy template (*f_ener_*) was the sum of the left and right smoothed activity patterns. In parallel, the auditory nerve activity patterns were cross-correlated, also in 10 ms segments. The binaural correlation template (*f_bc_*) consisted of the correlation coefficient between the two ears at lag 0, for each of the 80 segments of 10 ms composing the stimulus and for each frequency channel. Lag 0 is the lag at which the reduction in binaural correlation is largest in the N_0_S_π_ configuration, and is therefore the most informative lag.

Figure S1c (as well as S1d) shows the energy (left) and binaural correlation (right) templates for two words (same words used in Figure S1a & S1b). The energy template is essentially a smoothed version of the neural activity patterns. The binaural correlation template shows high correlation (close to 1) in those frequency/time combinations that are dominated by the noise (low-level energy activation). In frequency/time combinations that are dominated by the speech signal, the addition of the signal with reversed phase to the two ears causes a large decrease in binaural correlation. Thus, in the diotic configuration only *f_ener_* is informative (the binaural correlation template is identically 1 for all words). On the other hand, in the dichotic configuration, the correlation template *f_cc_* is different for different words, and therefore may be used to enhance the discrimination performance.

2. ***Making optimal decisions***

Since the external noise was frozen and was included in the generation of the templates, the only limitation on performance of the ideal listener comes from internal noise. Presumably, the internal noise is due to multiple sources in different levels of the nervous system; it is therefore modeled as a Gaussian noise. We assumed that the internal Gaussian noise was independent at different times, energy channels and correlation channels. Its variance had a fixed value at all energy channels and a different (fixed) value at all correlation channels.

For uncorrelated Gaussian internal noise, the statistically optimal decision in the case of consistent binaural protocol consisted of selecting the template that was closer (in the least squared difference sense) to the incoming sound [4]. In the mixed binaural protocol, the optimal decision required a more complicated decision variable, but which was still essentially based on the least squared difference distance between the incoming sound and the templates (see below). Thus, the main effort in the simulation consisted of determining the distance between an incoming sound and the templates. The actual performance depended on the amount of noise.

We used dynamic time warping (DTW [5,6]) to calculate the distance function between the input and the template words. The need for using DTW arose since regular Euclidian distance between the word templates did not correspond to the phonological similarity between them. Thus, using Euclidian distance, words which differed in a single phoneme (e.g. /barul/ - /parul/) were not necessarily closer to each other than words which differed in many phonemes (e.g. /dilen/ - /talug/). DTW is an algorithm that optimally warps the duration of acoustic segments in order to reach the smallest possible distance between the two words. Thus, for example, in Figure S1 the two phonologically-similar words /barul/ and /parul/ differ substantially when compared frame-by-frame throughout the nominal 800 ms duration. This is due to the fact that in Hebrew, voice onset time in /p/ occurs roughly at the stop release time while for /b/ there is substantial pre-voicing. Therefore, the word token for /barul/ contains a rather long initial closure period during which a voice bar is apparent. On the other hand, /parul/ essentially starts at voice onset time. As a result, the /arul/ part of /parul/ occurs earlier and is somewhat longer than the corresponding part of /barul/. Computing acoustic distance on a frame-by-frame basis would result therefore in a distance between /barul/ and /parul/ that is as large as the distance between two phonologically-different words, such as /dilen/ and /talug/. The DTW algorithm solves this problem by aligning the acoustically similar frames in the two words. As a result, most of the distance between the two words results from differences in the initial consonant (/b/ *vs*. /p/). Using the DTW metric therefore enables to simulate different templates of the same stimulus, with various temporal relations between their segments, all available to the optimal decision of the ideal listener.

The effect of the use of DTW on the distance between words is illustrated in Figure S1e, where the distance between phonologically-different (left) and phonologically-similar (right) word pairs is computed with and without the use of DTW. Whereas the acoustic, low-level distance is essentially the same when directly applying Euclidian distance to the word pairs, it is smaller for the phonologically-similar pair when the two words are aligned with DTW. Concomitantly, without DTW, the ideal listener predicted substantially better discrimination thresholds than the experimentally measured ones in the phonologically-similar cases, which did not fit well the measured thresholds. Note that although the DTW algorithm enabled a better fit of the thresholds, it did not affect the predicted binaural benefits, since it affected both diotic and dichotic thresholds similarly, compared to using a Euclidian metric.

In practice, given two time series X = {x_1_…x_N_} and Y = {y_1_…y_M_}, where each frame x_i_ and y_j_ is a length *k* vector, the DTW distance between them is the shortest distance between the two sequences, allowing the replication of frames (artificially lengthening the appropriate segment). The DTW distance is computed using dynamic programming: the function D(i,j) is calculated using the recursive formula

D(i,j) = d(i,j) + min([D(i-1,j), D(i-1,j-1), D(i,j-1)]),

where d(i,j) is the Euclidian distance between frames x_i_ and y_j_. This formula recursively computes the minimum distance between the subsequences {x_1_…x_i_} and {y_1_…y_j_}. The final DTW distance is then D(N,M).

In our case, X was the energy and correlation matrices calculated from the incoming input word, with the additional internal noise. Since each template in our simulation consisted of two matrices (*f_ener_* & *f_cc_*), we ran the algorithm on the combined distances for both energy and correlation templates, after normalizing their values by dividing each matrix by the variance of its corresponding internal noise. We computed the DTW distance between X and Y1 (template of word 1) and between X and Y2 (template of word 2) (for larger set sizes, see additional explanations below). The input word was then recognized as the word corresponding to the template with the smaller DTW distance. In the actual experiment, each word had two tokens (i.e., two recordings per experiment; see Materials & Methods). For each run of the simulation, one token of each of the two words was selected for generating the sequence of incoming word to be discriminated, and was then compared to all other tokens within the set.

The distance calculations were performed using only the channels X time combinations in which the SNR was larger than 0 dB when the overall SNR was 10 dB. Although the channels X times combinations to use were selected based on the energy templates, they were also used for the correlation templates.

The variance of the internal noise in the monaural channels was set to a value that fitted one of the experimental conditions (/barul/ - /parul/, used in Experiment II of Study 2, at -12 dB SNR where performance was 79.4%, measured under the consistent binaural protocol). Similarly, the variance of the internal noise in the binaural correlation channels was set to the value that yielded similar success rate in the dichotic configuration of the same words (~-22 dB SNR). These values were then used for **all** other conditions. The estimated thresholds for Study 1 (all experiments), Study 2 Experiment I and Study 2 Experiment II (phonologically-different word pair) were all computed with these estimates for the variances. Thus, for Study 1, we used the estimated variance values obtained from /barul/ - /parul/ to compute ideal listener performance for discrimination between all 4 word pairs used in Experiments I & III (/tamid/ - /amid/, /shalom/ - /chalom/, /chalom/ - /tamid/, /sikum/ - /amid/), under both consistent and mixed binaural protocols. In Experiment I of Study 2 we used the estimated variances to account for discrimination between 10 words (both phonologically-similar and phonologically-different; see main text), as well as between another pair of phonologically- different words (/arba/ - /tesha/). In Experiment II of Study 2 we used the same variances to calculate performance for the same pair (/barul/ - /parul/) under a different protocol (mixed) and under a different difficulty level (60% correct). Similarly, we calculated performance under the same conditions in this experiment for another pair of phonologically-different words (/dilen/ - /talug). We therefore used the variance values set from a single word pair in a single condition to explain thresholds obtained for a variety of word pairs, 10-word sets, different protocols and different difficulty levels.

### Results

**Study 1**

***Simulation results for the consistent binaural protocol (Experiment I)***

Although the two free parameters of the simulation were estimated using only a single word pair, both thresholds (Figure S2a & c) and binaural benefits (BILDs; Figure S2e) matched well the experimental ones in the consistent binaural protocol for both word pairs. The deviations between modeled and measured values were on average 1.5 dB (compare filled and empty bars of Figure S2e), while measured binaural benefits themselves were ~9 dB on average. Moreover, the dichotic thresholds were mainly determined by the change in correlation, with the energy channels below detection threshold (data not shown).

***Simulation results for the mixed binaural protocol (Experiment III)***

In the mixed binaural protocol, the ideal listener had two sets of templates for each word, one for the diotic configuration and one for the dichotic configuration. The optimal decision was not the least distance, but rather a complex function of the distances between the actual word that occurred and both templates. However, at the SNR levels relevant to the experiment, the templates for the diotic and for the dichotic configurations were so different that the optimal decision was well approximated by a sequential decision, in which the binaural configuration was detected first and then an optimal decision was performed using the appropriate templates for the selected binaural configuration.

Using the same *f_ener_* and *f_cc_* internal noise variances that were calculated for the consistent binaural protocol, the simulated results are essentially identical to the ones obtained for the consistent binaural protocol (see right side of Figure S2). This is in fact a proof that the suboptimal procedure used in the case of the mixed binaural protocol indeed reached optimal performance. The reason is that since the procedure used to determine these thresholds was suboptimal, these thresholds were only upper bounds – using the optimal procedure may have resulted in lower thresholds. However, the theoretical performance of an ideal listener in the mixed binaural protocol could not be better than its performance in the consistent binaural protocol, and therefore the thresholds in the consistent binaural protocol formed a lower bound on the thresholds in the mixed binaural protocol. Since the thresholds determined by the suboptimal procedure (upper bounds for the mixed binaural protocol) were essentially equivalent to those determined by the ideal listener in the consistent binaural protocol (lower bounds for the mixed binaural protocol), it follows that the use of the suboptimal procedure closely approximated the performance of the optimal procedure in the mixed binaural protocol.

***Summary of results of study 1***

The main result of this calculation is the finding of much larger binaural benefits than the ones actually obtained experimentally for phonologically-similar, but not for phonologically-different, word pairs in the mixed binaural protocol (compare filled and empty bars of Figure S2f; see Figure 1 of main text where these data are replotted). As can be seen in the figure, for the phonologically-similar pair, simulated binaural benefit is almost 7 dB larger than the measured one. This difference stems from discrepancies in dichotic thresholds, rather than diotic thresholds, between the simulation and the experiment. This result supports our claim that fully using the low-level information available should have resulted in higher binaural benefit values in the mixed binaural protocol.

**Study 2**

***Simulation results for Experiment I (perceptual load experiment)***

This experiment featured two conditions: “set size 2”, in which subjects were required to discriminate between pairs of words, and “set size 10”, in which subjects were required to discriminate between 10 words.

The “set size 2” condition of Experiment I, which required discrimination of pairs of words, was simulated similarly to Experiment III of Study 1, using the same preset estimates of the internal noise. Although the words used here were different from those used above, the simulation results matched nicely the phonologically-different set, but not the phonologically-similar one (Figure S3). For phonologically-different words, simulated binaural benefit differed from the experimental ones in ~0.4 dB. However, for the phonologically-similar pair, simulated binaural benefit was 3.7 dB larger than the one measured experimentally (Figure S3e); this difference stem from differences in dichotic thresholds for that pair (Figure S3c).

In the second experimental condition (“set size 10”, see main text), subjects were required to discriminate between 10, rather than 2, words. We simulated this condition by calculating the DTW distances between the input word and each of the 10 templates of the words comprising the set. The input word was then recognized as the template with the smallest DTW distance from the input word.

As in the case of set size 2, both simulated thresholds (Figure S3b & d) and binaural benefits (Figure S3f) matched the experimental ones only in the case of phonologically-different words. In case of phonologically-similar words (which consisted of 5 pairs of similar words, see Materials & Methods), simulated dichotic threshold was ~ 6 dB lower than the experimental one, resulting in a binaural benefit of 9 dB compared with the 3 dB measured experimentally. Simulated diotic thresholds were similar to the experimental ones.

***Simulation results for Experiment II (cognitive load experiment)***

This experiment required discrimination of pairs of pseudo-words, and was therefore simulated similarly to Experiments I & III of Study 1 (see above). The two conditions of the experiment yielded either 60 or 80% success. In the simulation, we matched the percent correct levels with the experimental ones; thus, the reported SNRs are the ones that yielded either 60 or 80 percent correct (Figure S4).

The results of the simulation in both percent correct levels matched nicely the experimental results of the phonologically-different pair, for both consistent and mixed binaural protocols (blue bars of Figure S4). Simulated diotic, as well as dichotic thresholds were similar to the experimental ones (Figure S4a-d). Binaural benefits (Figure S4e-f) were thus predicted to be large (10-11 dB) in all cases, as was indeed measured in the actual experiment (9-10.5 dB). The resultant maximal difference between simulated and experimental binaural benefits was therefore ~1.8 dB on average for the phonologically-different pair.

The simulation results also matched the ones obtained for the phonologically-similar pair, when measured under the consistent binaural protocol (red bars of Figure S4, left). Note, however, that this condition, discrimination between /barul/ and /parul/ in 80% success was the ones used for setting the 2 free parameters of the simulation, monaural and binaural variances. Thus, the fact that for 80% success thresholds and binaural benefits matched the experimentally measured ones is not surprising; however, the same values yielded similar results for the 60% success case as well, which was not used in the fitting process. In both cases, simulated binaural benefits fitted the measured ones, of about 9-10 dB.

In contrast, simulation results for the dichotic condition of the phonologically-similar pair differed substantially from the experimental ones when measured under the mixed binaural protocol, by an average of 5.6 dB for both 60 & 80% success (red bars of Figure S4d). As a result, the ideal listener simulations predicted substantially larger binaural benefits (9 & 10 dB for 60 & 80% success; Figure S4f) than the experimentally measured ones (3.2 & 4.6 dB, respectively).

***Summary of results of Study 2***

Similarly to Study 1, the simulation results of Study 2 show that in case of discrimination between phonologically-similar words under a mixed binaural protocol, an ideal listener obtains much larger binaural benefits than the ones measured experimentally. This trend is kept for both larger set sizes (Experiment I) and different percent correct levels (Experiment II).

### Summary & Conclusions

We used a standard simulation of the auditory nerve and SOC outputs, to simulate low representation levels of the auditory processing hierarchy. An ideal decision maker, with a DTW distance metric, was used to decide between the potential stimuli, with an internal Gaussian noise used as the source of limited performance. In order to set the variance of the internal noise in the monaural and binaural channels, we used a single simple case, of two phonologically-similar pseudo-words (/barul/ & /parul/) presented in a consistent binaural configuration, set to yield 80% success.

Given these two preset values for the internal noise, we were able to explain, using the model, many cases of the behavioral data. Thus, thresholds and binaural benefits for different word pairs, different protocols (consistent and mixed), different levels of difficulty (60 & 80% correct) and different vocabulary sizes (2 *vs*. 10) were all accounted for using the same parameters. On the other hand, the simulation, given these preset values, failed to account for dichotic thresholds (and hence binaural benefits) in conditions which involved discrimination between phonologically-similar words that were presented under the mixed binaural protocol and/or under conditions that required semantic processing (for which we could not use the simulation). The complex patterns of success and failure of the model, without changing any parameters from their initial values, fully matches the predictions of RHT, as explained in the main text.

**References**

1. Gresham LC, Collins LM (1998) Analysis of the performance of a model-based optimal auditory signal processor. J Acoust Soc Am 103: 2520-2529.

2. Bleeck S, Ives T, Patterson RD (2004) Aim-mat: the auditory image model in MATLAB. Acta Acustica 90: 781-788.

3. Bernstein LR, Trahiotis C (1996) The normalized correlation: accounting for binaural detection across center frequency. J Acoust Soc Am 100: 3774-3784.

4. Green DM, Swets JA (1966) Signal Detection Theory and Psychophysics. New York: Wiley.

5. Myers CS, Rabiner LR (1981) A comparative study of several dynamic time-warping algorithms for connected word recognition. The Bell System Technical Journal 60: 1389-1409.

6. Rabiner L, Juang B (1993) Time alignment and normalization. In: Cliffs E, editor. Fundamentals of speech recognition, Prentice Hall signal processing series. NJ: Prentice-Hall PTR. pp. 200-241.
